# Table Olive Fermentation Using Starter Cultures with Multifunctional Potential

**DOI:** 10.3390/microorganisms5020030

**Published:** 2017-05-28

**Authors:** Stamatoula Bonatsou, Chrysoula C. Tassou, Efstathios Z. Panagou, George-John E. Nychas

**Affiliations:** 1 Laboratory of Microbiology and Biotechnology of Foods, Department of Food Science and Human Nutrition, Agricultural University of Athens, Iera Odos 75, Athens GR-11855, Greece; m.bonatsou@aua.gr (S.B.); stathispanagou@aua.gr (E.Z.P.); 2 Institute of Technology of Agricultural Products, Hellenic Agricultural Organization—DEMETER, Sof. Venizelou 1, Lycovrissi, Attiki GR-14123, Greece; ctassou@nagref.gr

**Keywords:** table olives, probiotics, lactic acid bacteria, yeasts, fermentation, starter cultures

## Abstract

Table olives are one of the most popular plant-derived fermented products. Their enhanced nutritional value due to the presence of phenolic compounds and monounsaturated fatty acids makes olives an important food commodity of the Mediterranean diet. However, despite its economic significance, table olive fermentation is mainly craft-based and empirically driven by the autochthonous microbiota of the olives depending on various intrinsic and extrinsic factors, leading to a spontaneous process and a final product of variable quality. The use of microorganisms previously isolated from olive fermentations and studied for their probiotic potential and technological characteristics as starter cultures may contribute to the reduction of spoilage risk resulting in a controlled fermentation process. This review focuses on the importance of the development and implementation of multifunctional starter cultures related to olives with desirable probiotic and technological characteristics for possible application on table olive fermentation with the main purpose being the production of a health promoting and sensory improved functional food.

## 1. Microbiology of Table Olives Fermentation

Table olives are fermented fruits with a great impact on the economy worldwide and especially in the Mediterranean countries. Because of their high nutritional value and their content in fibers, amino acids, unsaturated fatty acids, vitamins and antioxidant compounds, table olives are considered to be an important functional food. The fermentation process provides preservation, enhanced nutritional and technological characteristics, as well as health benefits. Diverse microbial populations are involved in olive fermentation including members of lactic acid bacteria (LAB) and yeasts, which are the dominant species of the fermentation, as well as Enterobacteriaceae, *Clostridium*, *Pseudomonas*, and *Staphylococcus* and occasionally moulds [1]. These microorganisms and their metabolic activities determine the characteristics such as flavor, texture and safety [2]. Moreover, studies have shown that the microbiota of fermented table olives exhibits specific probiotic properties and thus olives are considered to be good matrices to deliver probiotics to the host while at the same time being free of lactose and cholesterol [3]. 

Table olive fermentation occurs spontaneously by the microbiota of the olive, without adding any starter culture. In general, the fermentation process is carried out by LAB and/or yeasts (Table 1). LAB are the main microorganisms responsible for the brine acidification by the production of lactic acid from fermentable substrates resulting to the decrease of the pH and provide microbiological stability to the final product as well as an extended shelf life. Yeasts on the other hand, mainly contribute to the determination of the aroma and taste due to the production of desirable metabolites and volatile compounds, while at the same time they enhance the growth of LAB and degrade phenolic compounds. Apart from the microbiological aspects of table olive fermentation, olive tree varieties could present different behaviors during brining because of the different fruit dimensions and physicochemical characteristics that would in turn affect the microbiota responsible for olive fermentation and influence the sensory profile of the final product [4]. 

However, spontaneous fermentations have many disadvantages compared to fermentations with starter cultures, as any deviations from the required conditions, such as the growth of undesirable strains and thereby metabolites can lead to an abnormal fermentation and a defective final product [21]. Inoculation of the brines with appropriate starter cultures of LAB or yeasts may reduce the risk of unexpected growth of spoilage strains and leads to a controlled and more stable fermentation process [22]. Moreover, studies have shown that using a starter in a small amount may accelerate the initial phase of fermentation resulting in desirable changes during the whole process [1], while at the same time it contributes significantly to the development of desirable structural and sensory characteristics [23,24,25]. Therefore, another beneficial effect of the inoculation with starter cultures is the production of a reproducible product of high quality [10,23,26,27,28]. Starter cultures are made of live microorganisms mainly isolated from fermented products which can be added during fermentation in a high cell number to accelerate and improve the fermentation process [29,30]. The most studied species for their potential use as starters in table olive production are LAB and yeasts. According to a recent study [31], the use of autochthonous yeasts and LAB has shown to shorten the time of fermentation, standardize the process, and improve their organoleptic and nutritional properties. Specifically, the role of microbial starters, selected for specific biotechnological and safety traits is considered to be the [31]:

(i) improvement of table olives sensory attributes;
(ii) control of the fermentation process;
(iii) monitoring of the correct evolution of the process;
(iv) maintenance and/or improvement of nutritional and healthy features of the product;
(v) protection from undesirable spoilage and pathogenic microorganisms;
(vi) fortification of table olives with microorganisms exhibiting probiotic potential;
(vii) enhancement of product stability and extension of shelf life.

## 2. Selection of Starter Cultures

However, the selection of specific strains displays important difficulties. The conditions during the fermentation process may be inhibitory for the growth of the selected strains. Moreover, the selection of inappropriate strains may lead to negative results such as the production of undesirable metabolites or even the diversion of the process. The selection and implementation of a starter culture is a crucial and complicated process for the success of the fermentation and although, there are no specific rules for the selection, there are three main steps to be followed [32]:

(i) isolation and “in vitro” selection; isolation from the fermentation environment and characterization of the strains is an important step for the selection;
(ii) validation on a laboratory scale;
(iii) validation at industrial scale; their characteristics and properties shown on the laboratory scale should be validated on large-scale fermentations.

Microorganisms should be easily adapted to the environment of the fermentation and have non-pathogenic, probiotic and technological characteristics. More specifically, the selected strains should have the ability to (i) decrease the pH value of the brine through their metabolic activity (production of specific organic acids), (ii) grow under the conditions of the fermentation (low pH, high salt concentration, low fermentation substrates), (iii) degrade phenolic compounds (oleuropein), (iv) produce desirable aroma and taste through the production of volatile compounds, (v) possess specific enzymes which contribute positively to the final product, and (vi) have probiotic, health promoting and disease preventing properties. In addition, their ability to grow and dominate the indigenous microbiota is a perquisite for their selection as starter cultures in olive fermentation. Although selected starter culture strains differentiate among various fermented foods, they should display some common characteristics. There are specific in vitro tests for the determination of these characteristics as shown in Table 2.

## 3. Probiotic Potential of Starter Cultures

The use of strains previously isolated from table olives, with probiotic and technological characteristics may lead to the production of a functional food which apart from its nutritional value provides a matrix for probiotic microorganisms. Microstructure of olives, in combination with their consistency of several components protects probiotic microorganisms during digestion. Moreover the ability of LAB to co-aggregate and form biofilms on the surface of olives creates new perspectives of table olives as a functional probiotic food.

Functional foods have no universally accepted definition. The term was first used in Japan in the 1980s when the Ministry of Health and Welfare initiated a regulatory system to approve certain foods with documented health benefits. Because of the fact that all foods provide taste, aroma and nutritive value, all foods can be considered as functional to some extent. However, foods are further studied for added physiological beneficial properties, which may optimize health [33]. An accepted and commonly used definition is that a functional food is a food given an additional function, often related to health-promotion or disease prevention by adding new ingredients or more existing ingredients. The claimed health benefits of fermented functional foods are expressed either directly through the interaction of ingested live microorganisms, bacteria or yeasts with the host (probiotic effect) or indirectly as a result of ingestion of microbial metabolites produced during the fermentation process (biogenic effect) [34]. A main field of functional foods is components called probiotics. According to the Food and Agriculture Organization of the United Nations and the World Health Organization (FAO/WHO) probiotics are defined as “live microorganisms which, when administered in adequate amounts, confer a health benefit on the host” [35]. The development of the probiotic concept is attributed to Metchnikoff, who observed that the consumption of fermented milks could reverse putrefactive effects of the gut microflora [36]. Probiotic foods are, in general, fermented foods containing live microorganisms in an adequate amount to reach the intestine. They contribute to the intestinal microflora balance of the host by the stimulation of the beneficial microorganisms and the reduction of pathogens [37,38,39]. To deliver the health benefits, probiotic foods need to contain an adequate amount of live bacteria (at least 10^6^–10^7^ colony forming units/g) [40,41]. Therefore, it is important to verify the presence of the inoculated probiotic starter culture in adequate numbers at the end of the fermentation process [42,43]. There are specific guidelines in order to claim that a food has a probiotic effect on the host. It is particularly important to know the genus and species of the probiotic microorganism. Α connection between the strain and a specific health effect should be observed after an accurate surveillance and an epidemiological study, implying that a probiotic effect is strain specific. There are several requirements to prove that a selected probiotic strain is safe for the host and it is of critical importance to assure safety, even among bacteria that are Generally Recognized as Safe (GRAS). Bacteria species which are generally recognized as safe are *Lactobacillus* spp., *Bifidobacterium* spp., *Streptococcus* spp., some *Enterococcus* and *Lactococcus lactis*. Moreover, *Saccharomyces cerevisiae* var. *boulardii* is the only yeast species recognized as safe.

The ability of LAB to colonize the surface of olives and the formation of biofilms during fermentation in combination with the microstructure of the olive surface that protects microorganisms during digestion establish new perspectives for the use of table olives not only as a nutritional fermented product but also as a probiotic carrier and hence a functional food with high added value [10,42,43,44,45]. There are some in vitro tests that determine the probiotic potential of strains and elucidate the mechanism of the probiotic effect, namely, gastric acidity and bile salt resistance, bile salt hydrolase activity, as well as, the ability to adhere on cell lines and exhibit antimicrobial activity and reduction or inhibition of pathogen adhesion to surfaces. However, available tests and in vitro data are not fully adequate and sufficient to predict the functionality of the strains in the human body. Human trials and clinical studies should be undertaken in order to confirm the applicability of probiotics in humans. The correlation between specific in vitro tests and in vivo data will be required and further validation with in vivo trials should be undertaken [35].

### 3.1. Resistance to Gastric Digestion and Pancreatic Digestion

In order to be characterized as potential probiotics, microorganisms “should be resistant to gastric juices and be able to grow in the presence of bile” [35]. Therefore, they should be resistant to the simulation of the conditions during gastric and pancreatic digestion and thus reach the intestine in an adequate amount in order to confer health benefits to the host. 

### 3.2. Auto-Aggregation/Co-Aggregation

Auto-aggregation and co-aggregation are of significant importance for probiotic strains as they are linked with their ability to adhere on epithelial cells. Moreover, co-aggregation may contribute to the inhibition of the colonization of pathogenic bacteria [46,47,48]. In addition, because of the hydrophobicity of microbial cells, aggregation may also inhibit biofilm formation of pathogens on surfaces [48,49,50]. 

### 3.3. Adherence to Mucus and/or Human Epithelial Cells and Cell Lines

The ability of both LAB and yeasts to adhere to intestinal mucosa is a crucial property for the exclusion of pathogens and undesirable bacteria. 

### 3.4. Antimicrobial Activity 

Possible antimicrobial mechanisms of specific microorganisms, for the control of pathogens are indicated below [35]:

- production of antimicrobial compounds
- production of bacteriocins
- competitive action on nutrients
- inhibition of binding due to competition
- formation of immune system

### 3.5. Biocontrol Agents

Some microorganisms act like biocontrol agents, because of their ability to produce substances such as ethanol and toxin proteins or glycoproteins, called killer factors. These factors can inhibit the growth of fungi and other undesirable microorganisms such as yeasts and bacteria [51,52]. 

### 3.6. Bile Salt Hydrolase Activity

Bile salt hydrolase (BSH) activity is considered to be an important property for probiotic strains. Several studies have shown that microbial BSH function in bile salts detoxification may contribute to the intestinal survival and persistence of producing strains and boost its survival in the hostile conditions of the gastrointestinal tract. Moreover, liberation of amino acids during bile salt deconjugation could be used as carbon, nitrogen, and energy sources and therefore provides a nutritional advantage on hydrolytic strains [53]. Bile salt hydrolase activity facilitates incorporation of cholesterol or bile into bacterial membranes and thus the increase in the tensile strength of the membranes [54,55,56]. These modifications on the cell surface from BSH activity could offer protection against perturbation of the integrity of the cell membranes by the immune system. Therefore, deconjugation of bile salts could lead to a reduction in serum cholesterol levels while at the same time may compromise normal lipid digestion, and the absorption of fatty acids [57]. 

### 3.7. Biodegradation of Phytic Complexes

Phytic acid is the main form of storage of phosphorous in mature seeds of plants and it is particularly abundant in olives. This acid, because of its ability to form chelates with metal ions such as calcium, iron and magnesium, creates insoluble complexes whose degradation requires specific enzymes. Because of the absence of these enzymes into the human gastrointestinal tract, the dephosphorylation of these complexes could be succeeded by microorganisms’ enzymes [52,58,59].

### 3.8. Biofilm Formation

Biofilm formation could also simultaneously enhance their viability during digestion. An important characteristic of starter cultures is their ability to adhere on the surface of the olive fruit [52,60]. The dominant species of the fermentation define the final characteristics of the olives. The dominance of LAB leads to a more acidic and stable product, whereas yeasts’ dominance results in a product with a milder taste. Because of the fact that the use of wild strains of yeasts and LAB exhibit diverse metabolic activity among the strains, the aroma and the taste of the final product are not consistent, while the quality is low [61]. For this reason, the use of starter cultures provides an enhanced and more predictable fermentation. Studies have shown that an appropriate starter culture implementation during the table olive fermentation contributes to a controlled and stable process, and thus the interest in using is continuously increased. The beneficial effect of using a starter culture in green table olives fermentation has already been observed for both Spanish and Greek varieties [23,26,27,62,63,64,65]. The use of two starters belonging to species *Lactobacillus plantarum* and *Lactobacillus pentosus*, contributed to a higher inactivation rate in comparison with the spontaneous fermentation [23]. The potential of biofilm formation of two multifunctional starters, namely, *Lactobacillus pentosus* B281 and *Pichia membranifaciens* M3A inoculated in a fermentation of Conservolea natural black olives was studied by [66]. *L. pentosus* and its ability to survive on the olive and form a biofilm with the indigenous microbiota in combination with its probiotic and technological properties makes it a possible starter for the production of a physicochemical and sensory acceptable final product with functional character. Moreover, despite the inability of *P. membranifaciens* M3A to recover at the end of the fermentation, the inoculation of the brine with the specific yeast strain resulted in the proper fermentation and a milder final product. The intake of probiotic strains from the consumption of table olives stimulates a protective immune response against pathogens and microbial balance into the intestine [38,67]. Probiotic strains may result in the microbial balance of the intestine through antagonistic activity against pathogens through the production of antimicrobial substances and the competition for nutrients [68,69,70]. In addition, they contribute to the improvement of nutritional status of the epithelium while at the same time decrease the permeability of intestine to toxic molecules [71]. A possible mechanism of the inhibition of the pathogenic strains is the inactivation of the enzyme urease which is responsible for the catalysis of the hydrolysis of urea to yield toxic metabolites like ammonia and plays an essential role in diseases such as yersiniosis, caused by the pathogen *Yersinia enterocolitica*. Seven strains belonging to *Lactobacillus* spp. isolated from olive brines were studied for their ability to adhere to Caco-2 cells, to tolerate low pH values and conditions that occur during digestion, as well as their antagonistic activity against the pathogenic bacterium *Yersinia enterocolitica* [72]. One strain, *L. paracasei* IMPC2.1 met the main criteria for probiotic potential, namely, inhibition of the pathogen, strong adhesion to Caco-2 cell as well as survival during simulated digestion. Moreover, another study has shown that another ureolytic human pathogen *Helicobacter pylori* was inhibited by the presence of the probiotic strain *Lactobacillus casei* [68].

Several studies have been undertaken in order to assess the probiotic potential of microorganisms related to table olive fermentation (Table 3).

## 4. Technological Characteristics of Starter Cultures 

Apart from their potential probiotic character, microorganisms implicated in table olive fermentation are considered to have important technological characteristics that affect the process one way or another as described below.

### 4.1. Εnhancement of Sensory Profile

Yeasts are known for their ability to produce different compounds such as ethanol, glycerol, higher alcohols, esters and other volatiles which contribute decisively to the formation of flavor while maintaining the texture of the olive fruit during fermentation [52,81,82]. 

### 4.2. Biodegradation of Phenolic Compounds

Oleuropein is the main phenolic compound of the olive fruit responsible for its bitter taste. In table olives processing, in order to degrade oleuropein at an acceptable level, olive fruits are subjected to chemical treatment with lye solution (NaOH). Many LAB and yeasts have the ability to biodegrade oleuropein during table olive processing by the production of β-glucosidase [82]. According to a recent study [83], 49 *Lactobacillus plantarum* strains isolated from dairy products and table olives showed a high degree of oleuropein degradation in modified MRS broth in which glucose had been substituted with 1.0% (w/v) of oleuropein; in particular strains Lp793, Lp790 and B51 hydrolyzed 93.6, 85.9 and 82% of oleuropein that corresponded to the presence in the medium of 101.5, 206.3 and 274.7 ppm of residual glucoside, respectively.

### 4.3. Ability to Grow in High Salt Concentrations

Natural black olive fermentation is taking place into brine containing 8–10% (w/v) NaCl, concentrations which could be an inhibitory factor for growth for several starters. For this reason, the ability of starters to grow in high salt levels could induce not only which will grow during the fermentation, but also the dominant species as well as the interactions among them [84]. Low salt concentrations could allow the growth of Gram negative bacteria leading to the diversion of the fermentation and the development of abnormal phenomena such as zapateria and alambrado.

### 4.4. Biofortification with Vitamins

Folic acid is the main co-factor for the biosynthesis of nucleotides. An adequate consumption of folic acid could reduce the risk of heart diseases and cancer. Yeasts follow the folic acid production biosynthetic path [52,58]. 

### 4.5. Reduction of Cholesterol

The increase of cholesterol levels in blood could be of great danger for the development of chronic heart diseases. Several microorganisms may contribute to the reduction of these levels due to their properties such as bile salt hydrolytic activity, bounding of their cells with cholesterol and cholesterol bioassimilation [53,54]. According to several studies, yeast strains isolated from table olive fermentations showed an ability to decrease cholesterol to a percentage higher than 60%, a fact that indicates that these strains could be used for a therapeutical purpose [52,85].

### 4.6. Production of Natural Antioxidants 

Microorganisms such as yeasts and LAB, as well as their extracts, have been recognized as a good source of antioxidants [86]. These natural antioxidants are presumed to be safer for humans and thus their use could lead to the replacement of synthetic compounds in food industry. Although the exact mechanism of their function requires more investigation, it is commonly agreed that they act by donating hydrogen protons to substrates and render them nonreactive to oxygen-derived free radicals [87,88,89]. Moreover, several yeast strains have shown to synthesize a number of bioactive compounds which can serve as antioxidants, such as carotenoids, tocopherols, citric and ascorbic acid [52,90,91].

### 4.7. Bio-Degradation and Bio-Assimilation of Mycotoxins

Detoxification activity occurs because of the detachment of the mycotoxins on the compounds of the cell wall [11,52,92]. As discussed above, yeasts could be used in order to decrease the levels of mycotoxins from both olive flesh and in the human intestine where the toxin have been absorbed.

### 4.8. Enhancement of Lactic Acid Bacteria Growth

The coexistence of yeasts and LAB as the dominant species during table olive fermentation is of a great importance. Yeasts can not only produce specific compounds such as vitamins, amino acids and purines but also hydrocarbons which are crucial for the growth of LAB. This synergistic symbiosis is particularly crucial for the development of a controlled and stable fermentation. 

### 4.9. Enzymatic Activity

The majority of the reactions taking place in live organisms cannot be performed at adequate rates without catalysis. The catalysts of the biological reactions are enzymes [93]. The study of the enzymatic activity is of great importance in order to select yeasts and LAB as starter cultures as these microorganisms can enhance or downgrade the quality of the raw material with the production of several metabolites and secondary compounds. As far as the table olives are concerned, enzymes could have both a positive and a negative effect on the quality of the final product. The two most commonly encountered enzymes are lipase and esterase that contribute to the formation of the aroma of the product. Because of the activity of these enzymes, an increase of free fatty acids is considered to be a precursor for the formation of several aromatic compounds. Lipolytic activity of these enzymes is a particularly desirable characteristic as during the catabolism of free fatty acids and the production of volatile compounds, the flavor and the organoleptic profile of the table olives is enhanced importantly [20,52,91,94].

Another important characteristic is the production of β-glucosidase that is related to the deconstruction of phenolic compounds and specifically of oleuropein. The activity of this enzyme leads to the debittering of olives, resulting in the reduced use of chemical compounds and proposes a more biological approach [30]. Alkaline phosphatase is a hydrolytic enzyme responsible for the subtraction of the phosphate group from various types of molecules such as nucleotides, proteins and alkaloids. The process of subtraction of the phosphate group is called dephosphorylation. During this process, inorganic phosphorus is released and becomes available, thus its transfer into the cells membranes is facilitated. In Gram negative bacteria, alkaline phosphatase exists in the periplasmic area, outside of the cell membrane. Lysine arylamidase is a proteolytic enzyme which catalyzes the hydrolysis of amino acids starting from the amino-terminal end of lysine of the polypeptide chain. Bacteria cannot directly assimilate the proteins and for this reason they depend on the extracellular and/or intracellular activity of these specific enzymes for the release of amino acids from polymers with high molecular weight. These amino peptidases are commonly isolated from microorganisms and plant tissues [95]. 

## 5. Conclusions

Table olives are considered by many food scientists as the “food of the future” owing to the healthy bioactive compounds they contain. Undoubtedly, the use of starter cultures for table olive fermentation becomes attractive for the food industry as it contributes to the reduction of cost, fermentation process time, risk of spoilage, as well as to the improvement of the process by enhancing the sensory characteristics of the final product and ensuring safety. Several strains belonging to *Lactobacillus* spp. have been successfully studied in pilot plan indicating their potential use during table olive fermentation processing for the production of a high added value functional product [96]. The future challenges will be to investigate more strains isolated from table olives for their probiotic and technological characteristics, in order to produce a health-related functional product with enhanced sensory characteristics.

## Figures and Tables

**Table 1:** Lactic acid bacteria and yeasts isolated from different table olive fermentation processing.

	Table Olive Preparations	Reference
*Lb. plantarum*	Spanish style green olives	[5]
	Spanish style green olives	[6]
	Untreated green olives	[7]
	Greek style black olives	[8]
*Lb. casei*	Untreated green olives	[9]
*Lb. brevis*	Untreated green olives	[9]
*Lb. pentosus*	Spanish style green olives	[10]
	Marketed olives	[11]
	Spanish style green olives	[6]
*Leuconostoc mesenteroides*	Spanish style green olives	[10]
	Marketed olives	[11]
*Pediococcus acidilactici*	Marketed	[11]
*Lb. coryniformis*	Spanish style green olives	[6]
*Lb. paraplantarum*	Untreated green olives	[7]
*Pediococcus ethanolidurans*	Greek style black olives	[8]
Yeasts		
*Aureobasidium pullulans*	Black Conservolea olives	[12]
*Candida aaseri*	Black Conservolea olives	[12]
*Candida apicola*	Cracked directly brined green olives	[13]
*Candida boidinii*	Seasoned green table olives	[13]
	Directly brine black olives	[14]
	Cracked directly brined green	[15]
	Directly brined green olives	[15]
	Black Conservolea olives	[12]
	Leccino olives	[15]
*Candida diddensiae*	Seasoned green table olives	[13]
	Cracked directly brined green olives	[15,16]
	Directly brined green olives	[17]
*Candida oleophila*	Cracked directly brined green olives	[15]
*Candida olivae*	Directly brined black table olives	[12]
*Candida parapsilosis*	Directly brined green table olives	[6]
*Candida quersitrusa*	Cracked directly brined green	[15]
*Candida silvae*	Black Conservolea olives	[12]
*Candida sorbosa*	Directly brined green	[8]
*Candida tropicalis*	Directly brined green Spanish style	[17]
*Candida tartarivorans*	Cellina di Nardo olives	[18]
*Citeromyces matritensis*	Cracked directly brined green	[15]
*Cystofilobasidium capitatum*	Black Conservolea olives	[12]
*Debaryomyces hansenii*	Black Conservolea olives	[12]
	Cellina di Nardo olives	[18]
	Black Conservolea olives	[19]
	Kalamata olives	[17]
*Debaryomyces ethcellsii*	Leccino olives	[18]
	Cracked directly Spanish style green olives	[14]
	Spanish style green olives	[17]
	Black Conservolea olives	[12]
*Issatchenkia occidentalis*	Seasoned green table olives	[12]
*Metschnikowia pulcherrima*	Black Conservolea olives	[12]
*Wickerhamomyces anomalus*	Directly brined black olives	[14]
	Black Conservolea olives; Directly brined green olives	[12]
	Cellina di Nardo olives	[18]
	Black Conservolea olives; Kalamata olives	[19]
*Pichia galeiformis*	Ripe black olives	[13,20]
	Cracked directly brined green olives	[16]
	Directly brined green olives	[17]
*Pichia guilliermondii*	Black Conservolea olives	[12]
*Pichia kluyveri*	Black Conservolea olives	[12]
*Pichia manshurica*	Black Conservolea olives	[12]
*Pichia membranifaciens*	Black Conservolea olives	[12]
	Directly brine green olives	[17]
	Cellina di Nardo olives	[18]
	Leccino olives; Kalamata olives	[19]
*Rhodotorula glutinis*	Processed black olives	[13]
*Rhodotorula graminis*	Processed black olives	[13]
*Rhodotorula diobovatum*	Black Conservolea olives	[12]
*Rhodotorula mucilaginosa*	Black Conservolea olives	[12]
	Cracked directly brined green olives	[15]
*Saccharomyces cerevisiae*	Processed black olives	[13]
	Seasoned green table olives	[12]
	Black Conservolea olives	[17]
	Directly brined green olives	[15]
	Cracked directly brined green olives	[17]
	Leccino olives; Kalamata olives	[19]
*Zygowilliopsis californica*	Black Conservolea olives	[12]
*Zygosaccharomyces mrakii*	Leccino olives	[18]
	Cracked directly brined green	[15]

**Table 2:** Desirable and undesirable characteristics of microorganisms used as starter cultures.

Properties	Desirable	Undesirable
Technological	Ability to survive/grow in different salt concentrations; Ability to survive/grow on high and low pH values; Ability to attach on olive epidermis	Production of CO_2_; Assimilation of lactic acid; Production of mycotoxins and biogenic amines
Probiotic	Survive in conditions simulating the passage through gastrointestinal tract; Ability to adhere and colonize the epithelial cells; Antimicrobial activity against pathogens; Ability to aggregate with pathogens	
Functional	Bio-assimilation of phenolic compounds such as oleuropein; Production of vitamins; Antimicrobial activity	
Enzymatic activity	Esterase; Lipase; Catalase; Phytase; Alkaline/acid phosphatase; β-glucosidase	Proteolytic and Xylanolytic activity

**Table 3:** Probiotic properties of lactic acid bacteria (LAB) and yeast species isolated from table olives.

Strains	Isolation Source	Probiotic Properties among Different Strains	References
*Lb. plantarum* ITM5BG, ITM4TG, ITM2TP, ITM4TP1 ITM5BG ITM5BG, ITM4TG, ITM2TP, ITM4TP1	Olive brines	High resistance to low pH and bile salts; Survival at simulated digestion; Adherence to Caco-2 cells	[73]
*Lb. plantarum* 16,19	Bella di Cerignola table olives	High resistance to low pH and bile salts; Adherence to IPEC-J2 cells; Inhibition of *E.coli* O157:H7 (for 19 only for the cells and the non-buffered supernatant)	[30]
*Lb. pentosus* B281, E97, E104	Fermentation brines of black olives; Mixed olives (black and green) brines	High resistance to low pH and bile salts; Adherence to Caco-2 cells	[74]
*Lb. plantarum* B282, E10, E69	Fermentation brines of black olives; Mixed olives (black and green) brines; Green olives brines	High resistance to low pH and bile salts; Adherence to Caco-2 cells	[74]
*Lb. paracasei* subsp. *paracasei* E93, E94	Mixed olives (black and green) brines	High resistance to low pH and bile salts; Adherence to Caco-2 cells	[74]
*Lb. pentosus* GG2S-T2-168, GM2F-T5-327	Fermentation brines os cv. Gordal, Manzanilla olives	High survival after gastric and pancreatic digestion; Autoaggregaton; Hydrophobicity	[75]
*Lb. plantarum* S11T3E, S1T10A, S2T10D S11T3E, S1T10A, S2T10D S11T3E S1T10A S11T3E, S2T10D	Nocellara Etnea green table olives	High resistance to simulated gastric digestion; Autoaggregation; Hydrophobicity; Increased transepithelial resistance of polarized H4-1 cells; Reduction in L. monocytogens invasion in gut model cells	[76]
*Lb. pentosus* S3T60C	Nocellara Etnea green table olives	High resistance to simulated gastric digestion; Autoaggregation; Inhibition of the adhesion Reduction in L. monocytogens invasion is gut model cells	[76]
*Lb. plantarum* 17.2b, B95, 69B, 607, P, FF28, 33	Portuguese cultivar/Campo Maior, Beja, Envendos	Survival at simulated digestion; Proteolytic activity; Adherence to Caco-2 cells; Exopolysaccharide production	[77]
*Lb. paraplantarum* B13, K, O1 B13, K, O1 B13, K, O1 B13 K, O1	Portuguese cultivar/Beja, Santarém, Ladoeiro	Survival at simulated digestion; Proteolytic activity; Adherence to Caco-2 cells; Hydrophobicity; Exopolysaccharide production	[77]
*Lb. pentosus* AP2-15N, CF1-39, CF2-10N, CF2-12, MP-10, AP2-16N, CF1-6, CF2-5, 5C2 CF2-10N, CF1-6, AP2-16N	Fermented Aloreña table olives	Survival under different gastric conditions Autoaggregation; Coaggregation with pathogens (*Listeria innocua*, *Staphylococcus aureus*, *Escherichia coli*, *Salmonella* spp.; Adhesion to intestinal cells; Antimicrobial activity against pathogens	[78]
*Lb. Plantarum* B282	Fermented olives of cv. Conservolea and Halkidiki	Adhesion on Caco-2 cells; Significant reduction of cancer cell proliferation	[79]
*Lb. Pentosus* B281	Fermented olives of cv. Conservolea and Halkidiki	Adhesion on Caco-2 cells; Significant reduction of cancer cell proliferation	[79]
*Pichia guilliermondi* Y16	Fermentation brines of naturally black cv. Conservolea olives	Survival in simulated digestion	[80]
*Pichia kluyveri* Y17	Fermentation brines of naturally black cv. Conservolea olives	Survival in simulated digestion	[80]
*Candida silvae* Y19	Fermentation brines of naturally black cv. Conservolea olives	Survival in simulated digestion; Extracellular phytase activity	[80]
*Metschnikowia pulcherrima* Y20	Fermentation brines of naturally black cv. Conservolea olives	Survival in simulated digestion; Intracellular and extracellular phytase activity	[80]
*Saccharomyces cerevisiae* Y22	Fermentation brines of naturally black cv. Conservolea olives	Survival in simulated digestion; Intracellular and extracellular phytase activity	[80]
*Pichia manshurica* Y37	Fermentation brines of naturally black cv. Conservolea olives	Survival in simulated digestion	[80]
*Rhodotorula mucilaginosa* Y38	Fermentation brines of naturally black cv. Conservolea olives	Survival in simulated digestion	[80]
